# AI-based HRCT quantification reveals DLCO and TLC as key determinants of ILD severity in connective tissue diseases

**DOI:** 10.1136/rmdopen-2025-005963

**Published:** 2025-10-28

**Authors:** Tobias Hoffmann, Ulf Teichgräber, Bianca Lassen-Schmidt, Diane Renz, Luis Benedict Brüheim, Tobias Weise, Martin Krämer, Joachim Böttcher, Felix Güttler, Gunter Wolf, Alexander Pfeil

**Affiliations:** 1 Department of Internal Medicine III, Friedrich Schiller University Jena, Jena, Thüringen, Germany; 2 Institute of Diagnostic and Interventional Radiology, Friedrich Schiller University Jena, Jena, Germany; 3 Fraunhofer Institute for Digital Medicine MEVIS, Bremen, Germany; 4 Department of Pediatric Radiology, Hannover Medical School, Hannover, Germany; 5 BioControl Jena GmbH, Jena, Germany

**Keywords:** Connective Tissue Diseases, Pulmonary Fibrosis, Lung Diseases, Interstitial

## Abstract

**Objective:**

Interstitial lung disease (ILD) represents the most common and severe organ manifestation observed in patients diagnosed with connective tissue diseases (CTDs). The aim of this retrospective cross-sectional study was to identify clinical risk factors such as pulmonary symptoms, age, gender, laboratory and pulmonary function test (PFT) parameters associated with the extent of ILD as measured by artificial intelligence-based quantification of pulmonary high-resolution computed tomography (AIqpHRCT).

**Methods:**

We included patients with a CTD-ILD diagnosis; all underwent PFT and HRCT, and pulmonary symptoms and signs of inflammation were also documented. AIpqHRCT was used to quantify lung volumetry and ILD features including ground glass opacities (GGO), reticulations, high-attenuation lung volume (HAV), emphysema and overall extent of ILD. Finally, 76 CTD-ILD patients were eligible for regression analysis, in order to evaluate the influence of clinical parameters on ILD extent.

**Results:**

The reduction of diffusing capacity of the lung for carbon monoxide (DLCO), total lung capacity (TLC) and elevated inflammation parameter was significantly associated with the extent of GGO, reticulations, HAV and overall extent of ILD. Pulmonary symptoms, age and forced vital capacity were not associated with the extent of ILD quantified by AIqpHRCT.

**Conclusion:**

The study presented that DLCO and TLC were predictive for the CTD-ILD severity. Consequently, our findings suggest the performance of PFT, including DLCO for all patients with CTD. In the case of reduced DLCO and TLC, further diagnostics, including HRCT, are necessary.

**WHAT IS ALREADY KNOWN ON THIS TOPIC**

- Interstitial lung disease (ILD) represents a well-recognised complication in patients with connective tissue diseases (CTD) and is associated with a significant morbidity and mortality. Artificial intelligence (AI)-based quantification of pulmonary high-resolution computed tomography (AIqpHRCT) is an established technique for the detection and quantification of CTD-ILD.

**WHAT THIS STUDY ADDS**

- In pulmonary function tests, diffusing capacity of the lung for carbon monoxide (DLCO) and total lung capacity (TLC) were associated with the CTD-ILD severity on quantitative evaluated HRCT. Pulmonary symptoms, age or gender were not associated with the extent of ILD quantified by AIqpHRCT.

**HOW THIS STUDY MIGHT AFFECT RESEARCH, PRACTICE OR POLICY**

- In our work, DLCO and TLC proved to be important parameters that determine the extent of ILD in CTD patients. In addition, AI-based automated analysis of HRCT data sets provides additional information that could be used more extensively in the future. Consequently, our findings suggest the performance of PFT, including DLCO, for all patients with CTD. In the case of reduced DLCO and TLC, further diagnostics, including HRCT, are necessary.

## Introduction

 Interstitial lung disease (ILD) is one of the most common and serious organ manifestations in patients with connective tissue diseases (CTD).^1 2^ The frequency of ILD differs between the CTD entities with the highest prevalence in patients with systemic sclerosis (SSc; 30.8%), mixed CTD (25.0%), and myositis (33.3%, dermatomyositis 100.0%).^1^ So far, the pathogenesis of ILD in CTD is not sufficiently explained. Usually, the diagnosis of ILD in CTD is based on the combination of clinical symptoms and physical examination, as well as non-invasive diagnostics such as pulmonary function test (PFT) and high-resolution computed tomography (HRCT). Regarding PFT, the forced vital capacity (FVC) is a known surrogate parameter for survival in CTD,^3 4^ but also the diffusing capacity of the lungs for carbon monoxide (DLCO) is already investigated for the early diagnosis of ILD in CTD.^2 5 6^ Total lung capacity (TLC) has only been studied marginally to date, as its measurement requires more specialised equipment. However, an association between this parameter and disease severity, as well as survival in SSc, has already been demonstrated.^7^

In clinical practice, interpreting symptoms, physical examination findings and HRCT is a skill that is based on expert knowledge. The situation is further complicated by the fact that only one-third of CTD patients with ILD present with pulmonary symptoms, which vary widely and range from dyspnoea, coughing and sputum production to finger clubbing.^5^ In addition, the visual (qualitative) evaluation of HRCT appears to be challenging, with several studies showing significant inter-observer variability even among experienced thoracic radiologists.^68^,^11^ Furthermore, the absence of a validated and established (quantitative) scoring system for HRCT has precluded any meaningful correlation with clinical parameters.

The deployment of new techniques such as artificial intelligence (AI)-based systems can facilitate the overcoming of such obstacles, thereby enabling the reliable quantification of the extent of ILD features observed in HRCT. The AI-based method for the quantification of ILD on HRCT (artificial intelligence-based quantification of pulmonary HRCT; AIqpHRCT) allows a quantification of different ILD features such as ground-glass opacities (GGO) or reticulations that is independent of the expertise of the reader. AIqpHRCT uses the browser-based platform SATORI (Segmentation and Annotation TOol for Radiomics and Deep LearnIng), showing already high performance in segmentation^6 12 13^ with automatic and reliable quantification and visualisation of ILD features based on each lung segment including the visualisation of the non-fibrotic and fibrotic ILD features.^6 14^ AI-based scoring systems also show moderate to strong correlations with visual scoring systems,^15^ but they can also predict mortality risk in idiopathic pulmonary fibrosis.^16^

Given that ILD is a leading cause of mortality in CTD—in patients with SSc 35% of the deaths are attributed to ILD^17^—early diagnosis and severity assessment as well as consistent therapy are essential. To improve the clinical understanding of ILD in patients with CTD, the aim of this study was to identify clinical risk factors such as pulmonary symptoms, age, gender, laboratory findings or PFT results which are associated with ILD extent as quantified by AIqpHRCT.

## Patients and methods

### Selection of patients

First, data from 100 consecutive patients with CTD-ILD treated in the Department of Internal Medicine III, Rheumatology and Osteology at the University Hospital Jena, Germany, between 2010 and 2022 were collected retrospectively . All patients fulfilled the classification criteria of one of the mentioned diseases.

The following inclusion and exclusion criteria had to be met:

#### Inclusion criteria

- The diagnosis of CTD-ILD, performed by a consensus panel of rheumatologists, pulmonologists and radiologists using a standardised pulmonary assessment based on clinical, laboratory, imaging and pathologic findings.
- Pulmonary HRCT, meeting the required minimum standards as mentioned below (see section HRCT).
- Availability of clinical parameters and PFT with forced expiratory volume per second (FEV1), forced vital capacity (FVC), total lung capacity (TLC), diffusing capacity of the lung for carbon monoxide (DLCO), and the diffusing capacity divided by the alveolar volume (DLCO/VA). PFTs were performed in our institution.

#### Exclusion criteria

- Patient with missing data in the context of the inclusion criteria.

An assessment of multicollinearity between the variables was performed. This was followed by a review of all HRCT scans in consensus (qualitative analysis) by two chest radiologists and one rheumatologist and a quantitative analysis of the most common pulmonary parenchymal ILD features in HRCT (each slice of all scans) using SATORI (AIqpHRCT) by a rheumatologist.

After applying inclusion and exclusion criteria, 76 patients were evaluable for regression analysis including the following items:

### Pulmonary Symptoms

Participants underwent a standardised pulmonary assessment including the quantification of pulmonary symptoms. The data was collected retrospectively.

- Pulmonary symptoms present: Dyspnoea, quantified according to the American Thoracic Society criteria,^18^ cough, sputum, bibasilar inspiratory crackles
- No pulmonary symptoms present

### Inflammatory parameters

Laboratory tests encompassed C-reactive protein (CRP; mg/dL), erythrocyte sedimentation rate after 1 hour (ESR; mm), and the lymphocyte count (GPt/L) at the timepoint of HRCT/PFT.

### PFT

PFT included FEV1, FVC, TLC, DLCO and DLCO/VA. Due to multicollinearity, FEV1 and DLCO/VA were excluded from the analysis. The predicted values of the PFT parameter were assessed by using reference values of the Global Lung Function Initiative (GLI).

### HRCT

Multi-slice CT was used for all HRCT images (General Electric Healthcare Technologies, Revolution, Waukesha, Wisconsin, USA) with a primary slice thickness of 0.625 mm (n=40) and 1 mm (n=36) and a reconstructed slice thickness of 2.5 mm or 3.0/4.0 mm. All scans were reviewed in consensus by two chest radiologists and a rheumatologist regarding parenchymal HRCT patterns (especially GGO, non-specific interstitial pneumonia (NSIP)*,* usual interstitial pneumonia (UIP) or other ILD patterns) according to the American Thoracic Society (ATS)/European Respiratory Society (ERS) and the Fleischner Society White Paper recommendations/criteria.^19^,^21^

### AI-based quantification of pulmonary HRCT (AIpqHRCT)

AIqpHRCT uses SATORI, a browser-based platform for curating medical data, developed by the Fraunhofer Institute for Digital Medicine MEVIS, Bremen/Germany.

The HRCT images were obtained from the hospital picture archiving and communication system and were pseudonymised using an in-house developed digital imaging and communications in medicine pseudonymisation platform before transferring into the RAdiological COOperative Network (RACOON) infrastructure. RACOON is a national research platform that provides and maintains a complete ecosystem for modern image-based medical research projects.^22^ Afterwards, the corresponding HRCT images were accessed via the web-based SATORI interface, and lung parenchymal changes were quantified by AIpqHRCT.^23^

In accordance with ATS/ESC and Fleischner Society White Paper recommendations/criteria,^19^,^21^ we used AIpqHRCT for an automated quantification of the following signs:

- GGO
- Reticulations
- Consolidations
- Honeycombing
- High-attenuation lung volume (HAV), a histogram-based measurement of lung fibrosis via Hounsfield units
- Overall extent of ILD as a parameter for the total lung involvement in ILD, defined as a sum of GGO patterns, reticulations, consolidations and honeycombing patterns

AIqpHRCT quantified automated lung volume (volumetry), GGO, reticulations, emphysema, HAV and overall extent of ILD. In addition, a manual segmentation of honeycombing was carried out in each of the (transversal) slices, performed by consensus of two rheumatologists, with both achieving >15 years and >5 years of experience in the field of HRCT evaluation. These quantitative data were then extracted from AIqpHRCT on an analysis/case basis and converted into an Excel file for further statistical analysis. All AIpqHRCT examinations were performed using a 2.5 mm or 3.0/4.0 mm slice thickness.

### Data analysis

Data collection and documentation were carried out using Microsoft Excel (Microsoft Windows, Redmond, Washington, USA). Descriptive data analysis and data processing were performed using the programming language Python (version 3.10.13) and the additional packages NumPy (version 1.26.0), Pandas (version 2.1.1), SciPy (version 1.11.4), and Statsmodels (version 0.14.0). Data visualisation was carried out using the packages Matplotlib (version 3.8.0) and Seaborn (version 0.13.0).

The variable ‘CRP’ exhibited a limited number of values that fell below the lower level of quantification (LLOQ=2 mg/L), which has been set to 1 mg/L (= LLOQ/2). Moreover, the variable ‘ESR 1 hour’ comprised a single value exceeding the upper level of quantification (ULOQ=120 mm/h), which was set to 121 mm/h (= ULOQ + LLOQ/2).

#### Multiple linear regression

The following AIpqHRCT variables were selected as outcomes of interest (dependent variables):

- Lung Volume [L]
- Emphysema [%]
- GGO [%]
- Reticulations [%]
- Overall Extent of ILD [%]
- HAV [%]

All dependent variables were log-transformed (numpy.log1p), aiming to achieve the characteristics of the normal distribution. In addition, all dependent variables were z-standardised in order to remove the influence of scale differences on the model parameters. Initial univariate analysis was performed, which was subsequently followed by a multivariate regression analysis that incorporated a select number of parameters.

The following variables were selected as predictors (independent variables) in univariate analysis:

- Gender [male/female, reference female]
- Age (years)
- Disease [myositis or CTD, reference CTD]
- Smoker [‘no’: 0, ‘ex’: 1, ‘yes’: 2]
- Symptomatic in general (reference no)—if one of the following symptoms was positive:
  - Dyspnoea according to the ATS criteria^18^
  - Bibasilar inspiratory crackles (reference no)
  - Cough (reference no)
  - Sputum (reference no)
- FVC [%]
- TLC [%]
- DLCO [%]
- NSIP (reference GGO)
- UIP (reference GGO)
- CRP [mg/dL]
- ESR 1 hour (mm)
- Lymphocyte count [GPt/L]

The variables CRP, ESR 1 hour and lymphocyte count were log-transformed (numpy.log1p). All independent variables considered continuous (age, ATS, smoker, CRP, ESR 1 hour, lymphocyte count, FVC, TLC and DLCO) were z-standardised.

Multicollinearity between the independent variables was quantified using variance inflation factors (statsmodels.stats.outliers_influence.variance_inflation_factor).^24^ All independent variables considered in multiple linear regression exhibited variance inflation factors<5.

**Figure 1:**
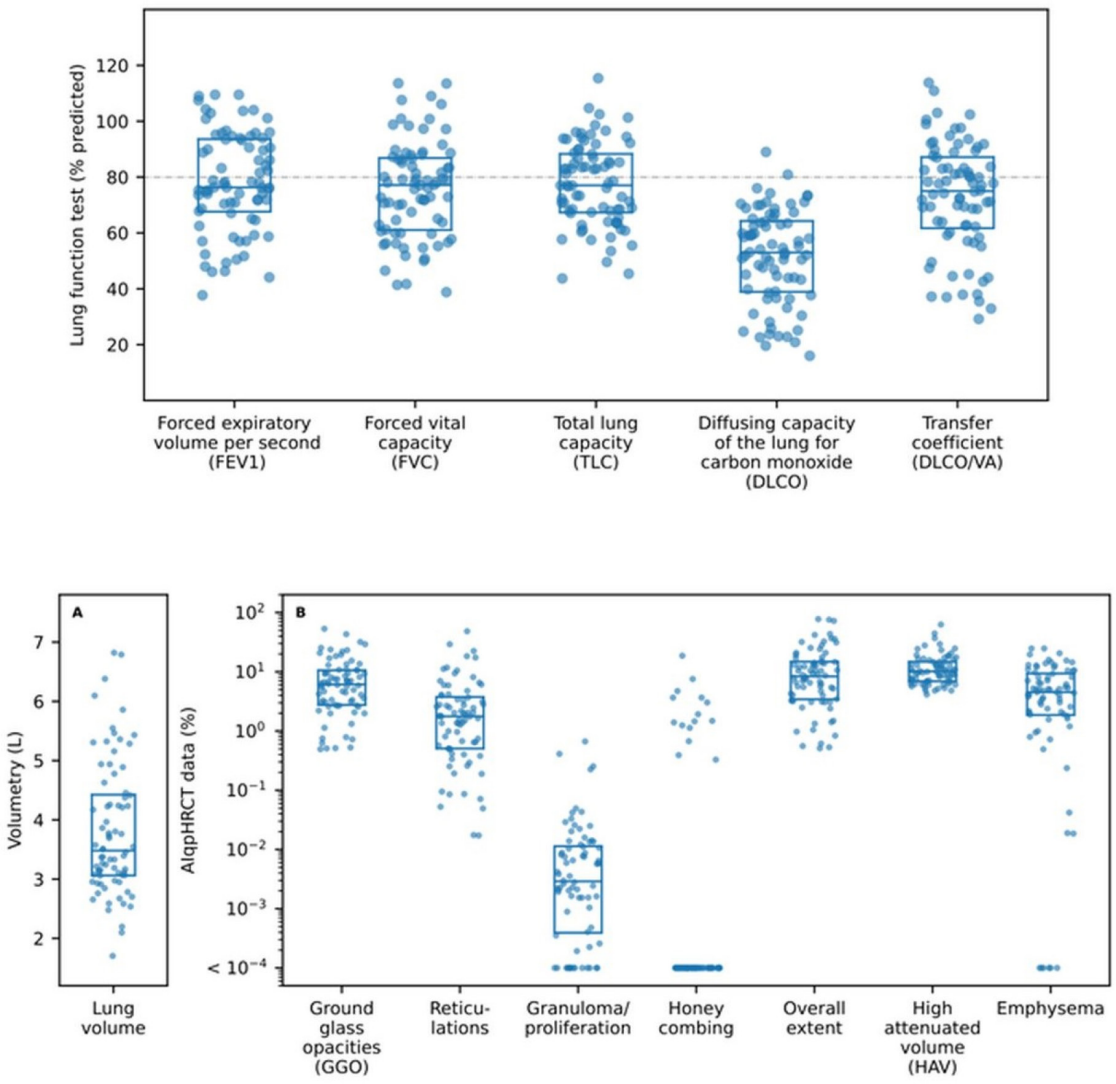
Box plots of the measured pulmonary function test (PFT) and AIqpHRCT data (reference line (---) demonstrates 80% predicted).

Multiple linear regression (statsmodels.formula.api.ols) was used to assess the independent associations between the dependent variables and selected independent variables considered in the analysis (see figure 1). Results were reported as effect coefficients (b) including their respective 95% confidence intervals (CI). P-values were reported to describe the significance of the respective independent variable’s influence on the respective dependent variable.

In the multiple linear regression analysis, we evaluated numerous clinical and laboratory parameters, including PFT, as independent variables. Due to multicollinearity, some parameters, especially FEV1 and DLCO/VA, were excluded. Moreover, the dependent variables “consolidations” and “honeycombing” were excluded, due to limited manifestations in the population. The multivariate regression analysis was performed in a stepwise manner, based on demographic data and the finding in univariate analysis. P-values<0.05 were considered as statistically significant.

## Results

### Baseline characteristics

This cross-sectional study encompassed 76 patients (22 men and 54 women) with CTD-ILD with a mean age of 59.0±13.2 years (see table 1). The most common CTDs were SSc (40.8%), followed by systemic lupus erythematosus (SLE; 15.8%), Anti-Jo1 syndrome (14.5%), dermatomyositis (10.5%), Sjögren’s disease (7.9%), mixed-connective tissue disease (MTCD; 6.6%), and polymyositis (3.9%). 64.5% of patients had a concurrent initial diagnosis of CTD and CTD-ILD.

**Table 1:** Baseline characteristics of the analysed CTDs-ILD patients

Baseline characteristics	Counts or mean value±standard deviation
**Number**	n=76
**Gender**	
Men	n=22 (28.9%)
Women	n=54 (71.1%)
**Age**	
Mean age±SD	59.0±13.2 years
**Initial diagnosis of CTD and ILD**	n=51 (67.1%)
**Disease duration in established disease (n=25)**	13.5±12.4 years
**Medication**	
No previous therapy	73.7% (n=56)
Azathioprine	14.5% (n=11)
Methotrexate	7.9% (n=6)
Mycophenolate mofetil	2.6% (n=2)
Cyclophosphamide	1.3% (n=1)
**CTD**	
Systemic sclerosis	40.8% (n=31)
Sjögren’s disease	7.9% (n=6)
Systemic lupus erythematosus	15.8% (n=12)
Mixed CTD	6.6% (n=5)
Dermatomyositis	10.5% (n=8)
Polymyositis	3.9% (n=3)
Anti-Jo1 syndrome	14.5% (n=11)
**Symptoms**	
Dyspnoea	n=47 (61.8%)
Cough	n=23 (30.3%)
Sputum	n=17 (22.4%)
Bibasilar inspiratory crackles	n=34 (44.7%)
No symptoms	n=17 (22.4%)
**Smoking**	
Active	n=10 (13.2%)
Ex-Smoker	n=16 (21.1%)
**Pulmonary function test**	
FEV1 in %	78.1±17.7
FVC in %	75.5±17.5
TLC in %	77.4±14.8
DLCO in %	51.6±16.9
DLCO/VA in %	72.9±19.5
**Laboratory tests**	
C-reactive protein; mg/dL	16.9±26.5
Erythrocytes sedimentation rate; mm	29.3±23.8
Lymphocyte count; GPt/L	1.29±0.73
**Distribution of ILD**	
Ground-glass opacities	n=32 (42.1%)
Non-specific interstitial lung disease	n=36 (47.4%)
Usual interstitial lung disease	n=8 (10.5%)

CTD, connective tissue disease; DLCO, diffusing capacity of the lung for carbon monoxide; DLCO/VA, diffusing capacity divided by the alveolar volume; FEV1, forced expiratory volume per second; FVC, forced vital capacity; ILD, interstitial lung disease; TLC, total lung capacity.

### Pulmonary symptoms

The most relevant symptoms were dyspnoea in 61.8% and bibasilar inspiratory crackles in 44.7% of patients with 22.4% being asymptomatic. 13.2% of the patients were active and 21.1% ex-smoker.

### Inflammatory parameters

At baseline, mean values for CRP were 16.9±26.5 mg/dL, ESR 1 hour 29.3±23.8 mm, and lymphocyte count 1.29±0.73 Gpt/L lymphocytes.

### PFT

FEV1 and FVC showed a mean value of 78.1±17.7% and 75.5±17.5%, respectively. Mean values for TLC were 77.4±14.8%, for DLCO 51.6±16.9%, and DLCO/VA 72.9±19.5% (see table 1 and figure 1).

**Figure 2:**
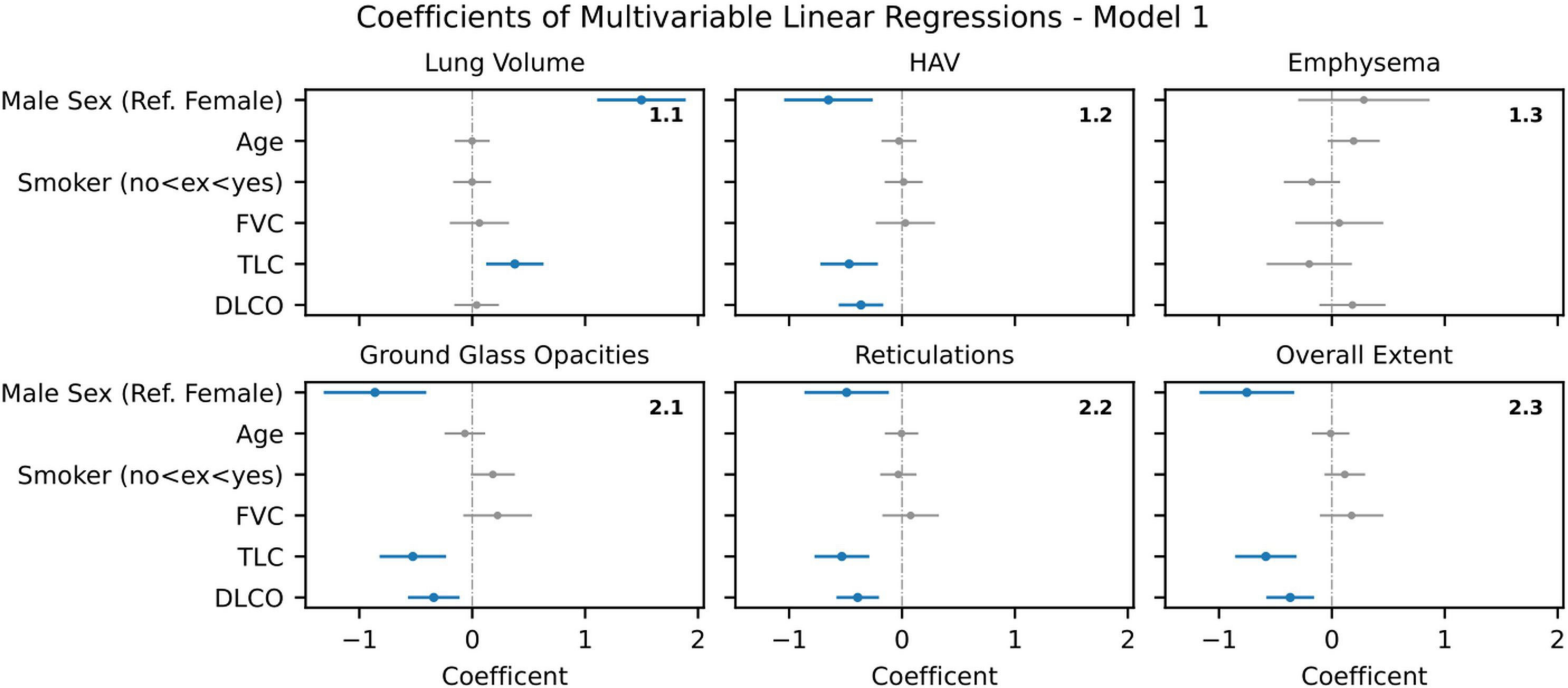
Model 1 – Multiple linear regression analysis of AIqpHRCT data with demographic data and PFT (blue-marked parameters with significant effect in regression analysis). AIqpHRCT, artificial intelligence-based quantification of pulmonary high-resolution computed tomography; DLCO, diffusing capacity of the lung for carbon monoxide; FVC, forced vital capacity; HAV, high-attenuation lung volume; PFT, pulmonary function test; TLC, total lung capacity.

### HRCT

42.1% (n=32) of the patients showed pure GGO, 47.4% (n=36) NSIP and 10.5% (n=8) UIP in HRCT scans (see table 1).

### AIpqHRCT

The quantitative analysis of AIqpHRCT revealed 3.81±1.13 L in volumetry and 6.13±5.87% emphysema. Regarding ILD, the analysis showed 8.91±9.66% GGO, 4.06±7.33% reticulations, 0.03±0.09% consolidations and 0.68±2.41% honeycombing. In addition, AIpqHRCT demonstrated 12.70±9.37% HAV and 13.60±16.20% overall extent of ILD (see table 2 and figure 1).

**Table 2:** Results of the artificial intelligence-based quantification of pulmonary HRCT (AIqpHRCT) in CTD-ILD patients

Parameter	Mean value±SD
Volume; L	3.81±1.13
Ground-glass opacities; %	8.91±9.66
Reticulations; %	4.06±7.33
Consolidations; %	0.03±0.09
Honeycombing; % *	0.68±2.41
Overall extent of ILD; %	13.60±16.20
High attenuation volume (HAV); %	12.70±9.37
Emphysema; %	6.13±5.87

* With operator adjustment.

### Univariate regression analysis

In the univariate analysis, we examined the influence of the independent variables on AIqpHRCT data. In particular, the PFT parameters (FVC, TLC and DLCO) correlated with the AIqpHRCT data collected (HAV, GGO, reticulations and overall extent of ILD), but also with the patients symptoms (especially dyspnoea, bibasilar inspiratory crackles and the symptomatic in general) and elevated CRP. The results are presented graphically and tabularly in the online supplemental figure 1 and table 1.

Based on the findings and in consideration of pre-existing data, the development of three models was initiated in a sequential manner. Since demographic data such as gender, age and smoking status can have a known influence on ILD, these were considered in each model. Multicollinearity, especially between the PFT parameter (FVC, TLC and DLCO), was quantified and excluded.

### Multiple linear regression analysis for the prediction of ILD in CTD

#### Model 1 – Demographic data and PFT

Significant correlations were consistently found for the independent variables gender, TLC and DLCO with the dependent variables HAV (b=−0.65 [-1.04,–0.26], p=0.001; b=-0.47 [−0.72 to –0.21], p<0.001; b=−0.36 [-0.56,–0.17], p<0.001), GGO (b=-0,86 [−1.32 to –0.41], p<0.001; b=−0.53 [-0.82,–0.23], p=0.001; b=-0.34 [−0.57 to –0.11], p=0.004), reticulations (b=−0.49 [-0.86,–0.12], p=0.011; b=-0.53 [−0.78 to –0.29], p<0.001; b=−0.39 [-0.58,–0.20], p<0.001) and overall extent of ILD (b=−0.75 [-1.17,–0.33], p=0.001; b= -0.58 [−0.86 to –0.31], p<0.001; b=−0.37 [-0.58,–0.16], p=0.001). No significant correlation was found for age, smoking status or FVC. For lung volume, regression analysis revealed significant correlations with gender (b=1.50 [1.11, 1.89]; p<0.001) and TLC (b=0.38 [0.12, 0.63]; p=0.004). (see figure 2)

#### Model 2 - Demographic data, PFT and pulmonary symptoms

**Figure 3:**
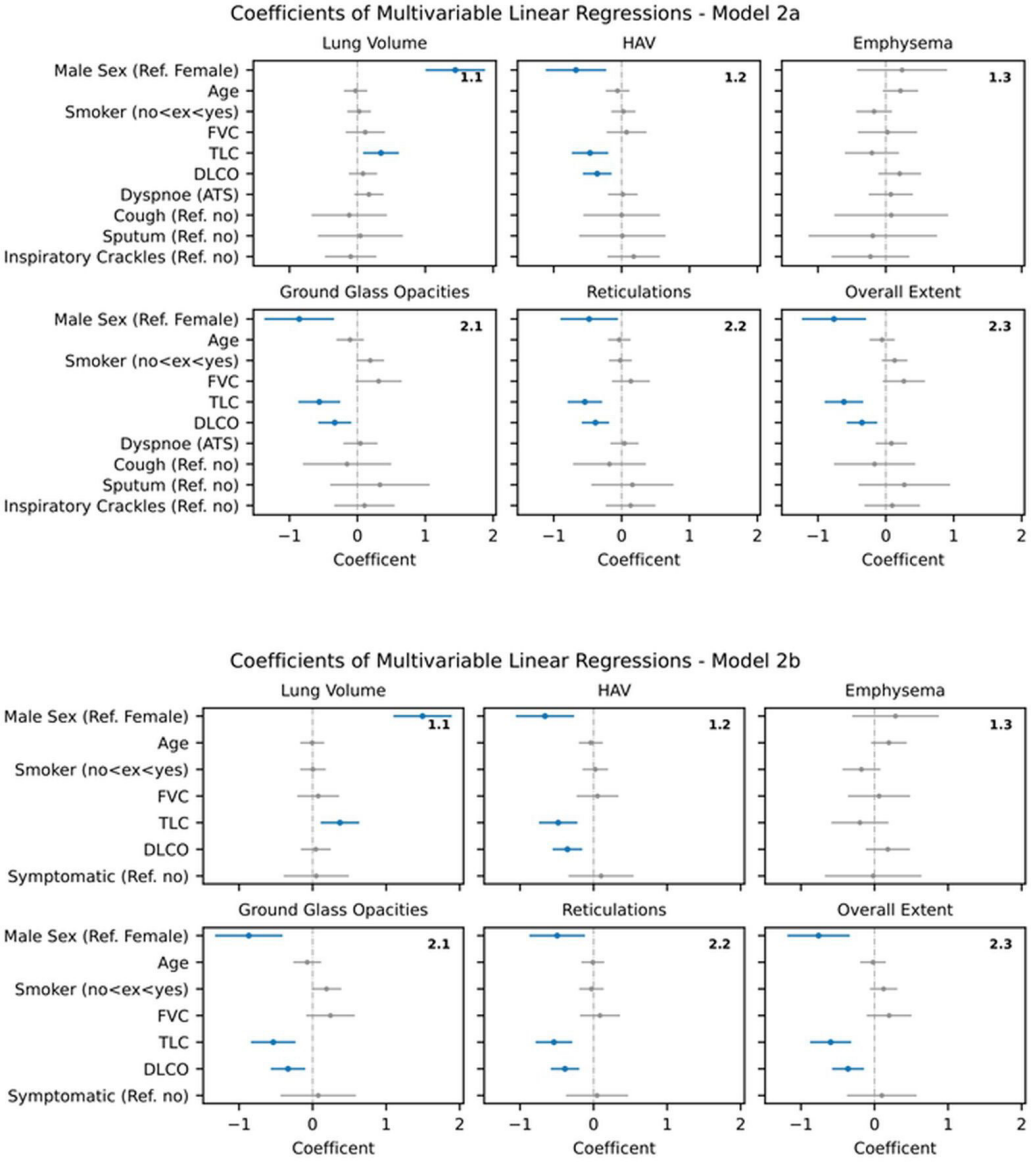
Model 2 a/b – Multiple linear regression analysis of AIqpHRCT data with demographic data, PFT and pulmonary symptoms (blue -marked parameters with significant effect in regression analysis). AIqpHRCT, artificial intelligence-based quantification of pulmonary high-resolution computed tomography; DLCO, diffusing capacity of the lung for carbon monoxide; FVC, forced vital capacity; HAV, high-attenuation lung volume; PFT, pulmonary function test; TLC, total lung capacity.

When pulmonary symptoms (eg, dyspnoe, cough, sputum, sclerophonia) were added, no relevant changes to Model 1 were observed; in particular, no significant correlations were found between pulmonary symptoms and any AIqpHRCT parameter. Even when using the combined variable (symptomatic in general), no significant correlation with the AIqpHRCT data could be demonstrated. Despite the attainment of significant results in the univariate analysis for pulmonary symptoms, the effect was not sufficiently robust in the multivariate analysis. (see figure 3a,b)

#### Model 3 - Demographic data, PFT, pulmonary symptoms and CRP

**Figure 4:**
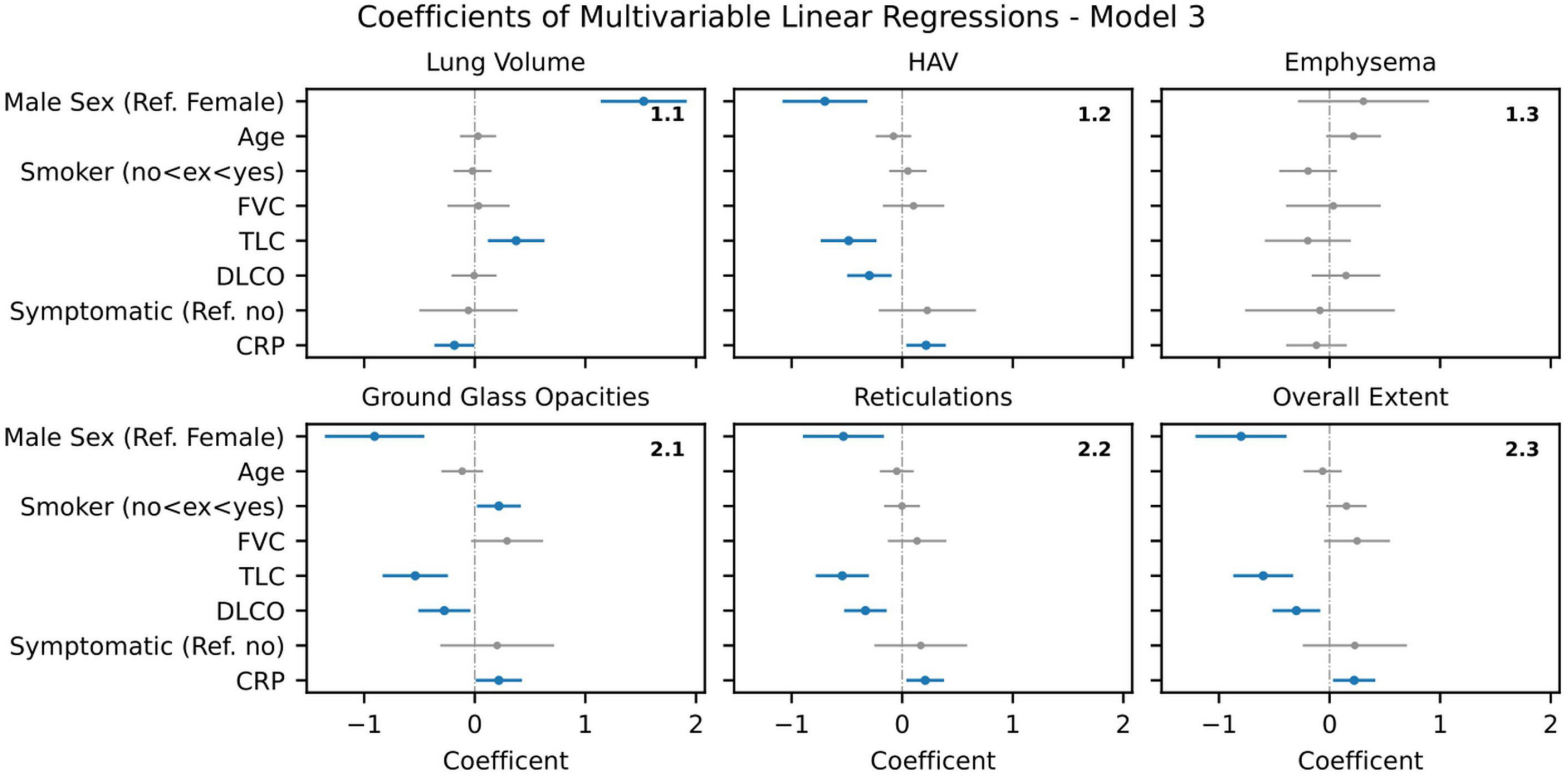
Model 3 – Multiple linear regression analysis of AIqpHRCT data with demographic data, PFT, pulmonary symptoms and CRP (blue -marked parameters with significant effect in regression analysis). AIqpHRCT, artificial intelligence-based quantification of pulmonary high-resolution computed tomography; CRP, C-reactive protein; PFT, pulmonary function test.

In Model 3, CRP was added, but this did not result in any relevant changes to the previously collected data. Significant associations continued to be found for the AIqpHRCT data (HAV, GGO, reticulations, overall extent of ILD) with gender, TLC and DLCO. CRP correlated positively with HAV (b=0.22 [0.04, 0.39], p=0.018), GGO (b=0,22 [0.01, 0.43], p=0.041), reticulations (b=0.21 [0.04, 0.38], p=0.017), overall extent of ILD (b=0.22 [0.03, 0.41], p=0.023) and negatively with lung volume (b=−0.18 [-0.36, 0.00], p=0.045). There was still no significant correlation with the pulmonary symptoms (see figure 4).

Regarding the significant effect of gender on the extent of GGO, HAV and overall extent of ILD, secondary analysis for the effect/bias of other baseline characteristics on gender was performed, but without significant results in disease distribution (p=0.085), age (p=0.080) or pulmonary symptoms (p=0.962). However, there were significantly fewer smokers in the female population (p=0.010).

## Discussion

**Figure 5:**
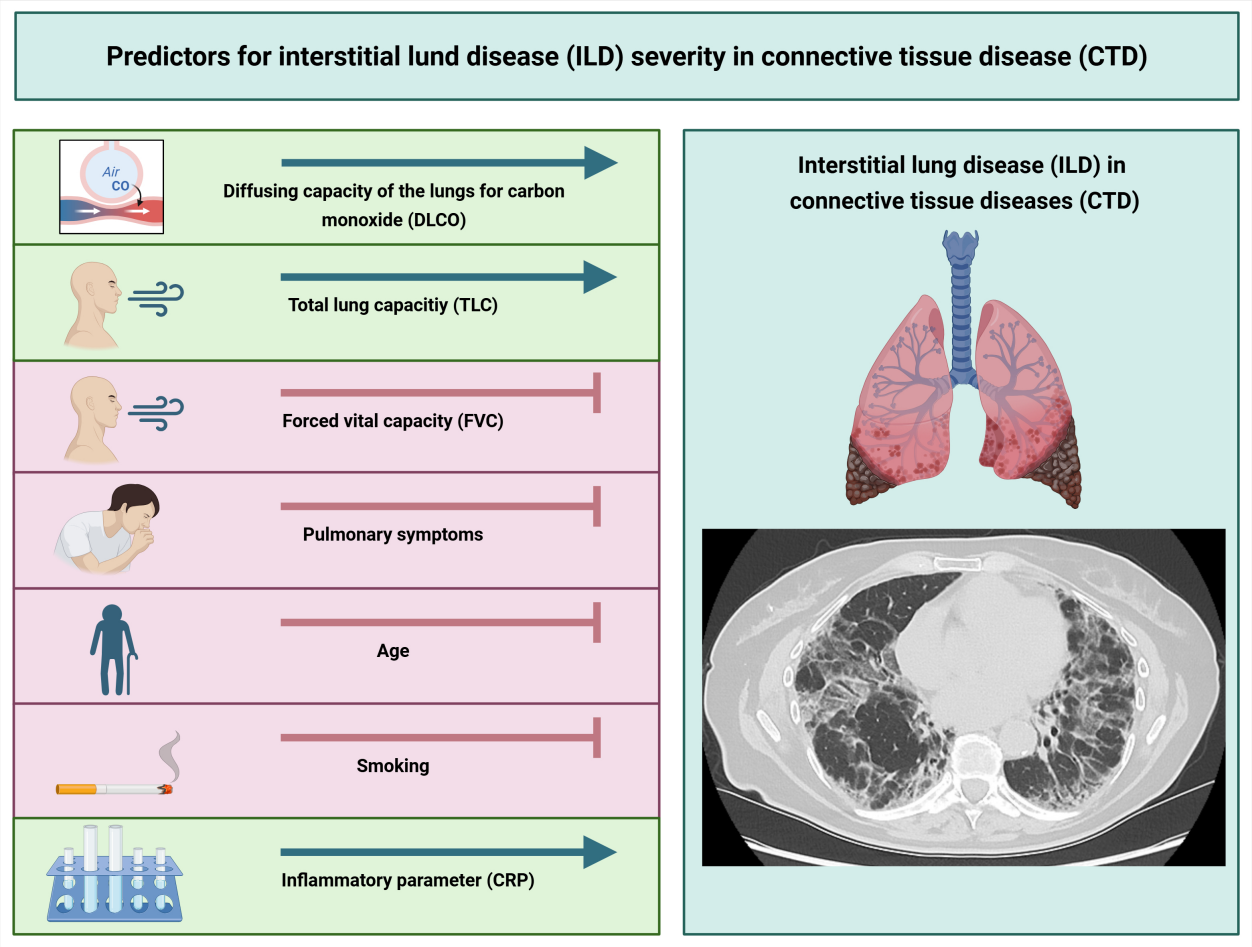
Predictors for interstitial lung disease (ILD) severity in connective tissue diseases (CTD) (Created in BioRender. Pfeil, A. (2025) https://BioRender.com/sm3t9n5).

Depending on the disease, ILD is one of the most common and serious organ manifestations in CTD with an increased mortality. In this context, it is of clinical importance whether pulmonary symptoms, age, gender, laboratory findings or PFT results have a predictive value for the prediction of CTD-ILD. In this context, we performed a retrospective analysis to evaluate the effect of several clinical, laboratory and PFT parameters on quantified ILD features in patients with CTD-ILD using AIqpHRCT (see figure 5).

### PFT parameters

The PFT results in our study revealed slightly reduced FEV1, FVC and TLC; only DLCO as a parameter for diffusing capacity of the lungs was significantly reduced with a mean value of 51.6±16.9%. Current recommendations emphasise the need for screening in CTD patients, based on risk factors, by using HRCT and monitoring disease progression by using HRCT and PFT.^25^ Nevertheless, there is an absence of international consensus on the definitions of disease severity, progression and outcome for CTD-ILD. Among PFT parameters, FVC decline is a commonly used primary endpoint in therapeutic trials in CTD-ILD.^3 4^ Our study highlighted that the severity of all ILD features (GGO, reticulations, overall extent of ILD and HAV) demonstrated a significant negative association with parameters of PFT, including TLC and DLCO, but not with FVC. Nevertheless, this supports arguments against a significant negative correlation and consequently against FVC as a parameter for predicting ILD, even if its use is unavoidable in the assessment of ILD prognosis.^3 4^ In literature, there are also studies that demonstrate the efficacy of TLC as a predictor of disease severity and survival in SSc.^7^ Tashkin *et al* demonstrated in data of the scleroderma lung study I and II a significant correlation in FVC, TLC and DLCO, but in multivariate regression analysis, DLCO provides the best estimation of texture-based ILD measurement on HRCT; FVC instead was not a good parameter for predicting the extent of ILD.^26^ There are also several other studies demonstrating the correlation between PFT parameters and quantitative and AI-based HRCT analysis.^6 15 27 28^

The rationale behind the limited value of FVC in predicting the severity of CTD-ILD, as demonstrated by studies conducted by us and other researchers, remains to be explained. In this context, Handa *et al* demonstrated a stronger correlation for FVC with the bronchial volume (φ=−0.617, p<0.001) than to extent of ILD (=−0.514, p<0.001) in IPF patients, with the opposite effect observed for DLCO.^15^ Nevertheless, FVC is a relevant prognostic factor in CTD-ILD, but it cannot adequately reflect damage peripheral to the bronchioles. Accordingly, a study by Hoesein *et al* demonstrated in patients with chronic obstructive pulmonary disease, through quantitative lung imaging, that FVC is particularly associated with airway wall thickness, while TLC is associated with emphysema.^29^

In consequence, the quantification of DLCO and TLC is essential for the detection,^5^ monitoring^25^ and severity assessment of CTD-ILD and all patients with CTD should require a PFT including DLCO for the detection of CTD-ILD, while FVC is not a relevant parameter in predicting ILD severity. In the case of reduced DLCO, further diagnostic workup, including HRCT^5^ and bronchioalveolar lavage for differential diagnosis, is necessary.^2 30^

### Clinical and laboratory parameters

Our study revealed only in univariate analysis a significant association between pulmonary symptoms and the presence of CTD-ILD. In previous data, our group was able to show significant differences between symptomatic and asymptomatic patients with initial diagnoses of both ILD and CTD.^31^ In multivariate analysis, considering other confounding factors (especially PFT), pulmonary symptoms do not provide a more reliable identification of CTD-ILD in this study. There is a need for further prospective studies, investigating the effect of systematic recording of symptoms for the prediction of presence or extent of ILD.

In contrast, CRP demonstrated in both univariate and multivariate analysis a positive association with HAV, GGO, reticulations and overall extent of ILD. These findings are concordant with available literature, demonstrating that elevated inflammation marker (eg. CRP) is associated with increased risk of ILD in SSc, idiopathic inflammatory myopathy or Sjögren’s disease,^25 32 33^ but also with prognosis/long-term progression.^34 35^

Furthermore, the study demonstrated a substantial increase in lung volume in male subjects and those with higher TLC. These are well-known physiological facts and confirm the correct measurement of lung volume, as this is used to calculate the proportions of ILD.

In contrast to volumetry, we demonstrated significantly higher levels of GGO, overall extent of ILD and HAV in female patients. For an appropriate interpretation of this result, secondary analysis of the effect of gender on other baseline characteristics was performed, but without significant effects in disease distribution, age or pulmonary symptoms. However, there were significantly fewer smokers in the female population. In general, it is known that male gender has a higher risk of ILD development and progression, especially in SSc or myositis.^32 36^ However, in our study, female patients had a higher incidence of ILD at initial diagnosis. The underlying cause of this phenomenon remains to be clarified, although one potential explanation may relate to a heightened level of awareness regarding ILD in male subjects, but further studies should investigate this effect. Furthermore, it should be noted that selection bias could play a significant role. Conversely, the overall risk of autoimmune diseases is elevated in women compared with men,^37^ resulting in a greater number of female patients with CTD-ILD.

### HRCT data analysis

Regarding the qualitative analysis of HRCT, we have already been able to demonstrate similar results in previous studies.^1 2 5^ As usual in CTD, the proportion of NSIP (47.4%) was clearly higher compared with the UIP pattern (10.5%).^38 39^ Further, AIpqHRCT demonstrated an involvement of lung parenchyma in CTD-ILD as quantified by overall extent of ILD with 13.60±16.20% and HAV (extent of lung fibrosis) with 12.70±9.37%. Moreover, Saldana *et al* demonstrated similar results with a HAV of 7.5% (CI 5.2 to 10.6%) in patients with SSc-associated ILD.^40^

The strength of our study is the evaluation of clinical parameters as predictors for CTD-ILD extension as quantified by AIqpHRCT. Limitations of this study are the monocentric and retrospective design, but also the limited number of CTD-ILD patients and potential selection bias. Even if patients are examined in a structured manner and the indication for HRCT is made according to current guidelines, it cannot be ruled out that patients with poorer PFT (DLCO, FVC or TLC) were more likely to receive imaging, resulting in bias.

In order to generate more knowledge about CTD-ILD, we would recommend prospective multicentre studies with quantitative HRCT analysis be conducted. However, the direct implications for clinical routine remain unclear at this time. PFT is and remains an indispensable part of ILD diagnostics. It can provide additional, functional information that HRCT cannot provide. We recommend future combination of quantitative HRCT analysis and PFT, but further research is needed here.

## Conclusion

ILD is a common and severe complication in CTD patients and a significant risk factor for increased mortality. Based on the quantification of ILD through AIpqHRCT, we were able to identify risk factors of ILD severity in CTD patients. Pulmonary symptoms, age or gender were not associated with ILD severity as quantified by AIqpHRCT. In addition, FVC, a known surrogate for ILD prognosis, was not a predictor of CTD-ILD severity. In contrast, the PFT parameters DLCO and TLC were found to be associated with CTD-ILD extent. As a clinical consequence, for all patients with CTD, a PFT should be performed, including DLCO. In the case of reduced DLCO and TLC, further diagnostics, including HRCT, are necessary. Consequently, AI-based HRCT analysis offers the potential to make reliable statements regarding the extent of ILD and to investigate associations with established clinical parameters. The potential of this method, to be used in the quantification of therapeutic responses in HRCT, is worthy of further investigation.

## Supplementary material

10.1136/rmdopen-2025-005963 online supplemental figure 1

10.1136/rmdopen-2025-005963 online supplemental file 1

## Data Availability

Data are available upon reasonable request.
