# Supplementary figures and images for: AI-based HRCT quantification reveals DLCO and TLC as key determinants of ILD severity in connective tissue diseases
Source: RMD Open. 2025 Oct 28;11(4):e005963. doi: 10.1136/rmdopen-2025-005963 (PMC12570930; doi:10.1136/rmdopen-2025-005963)

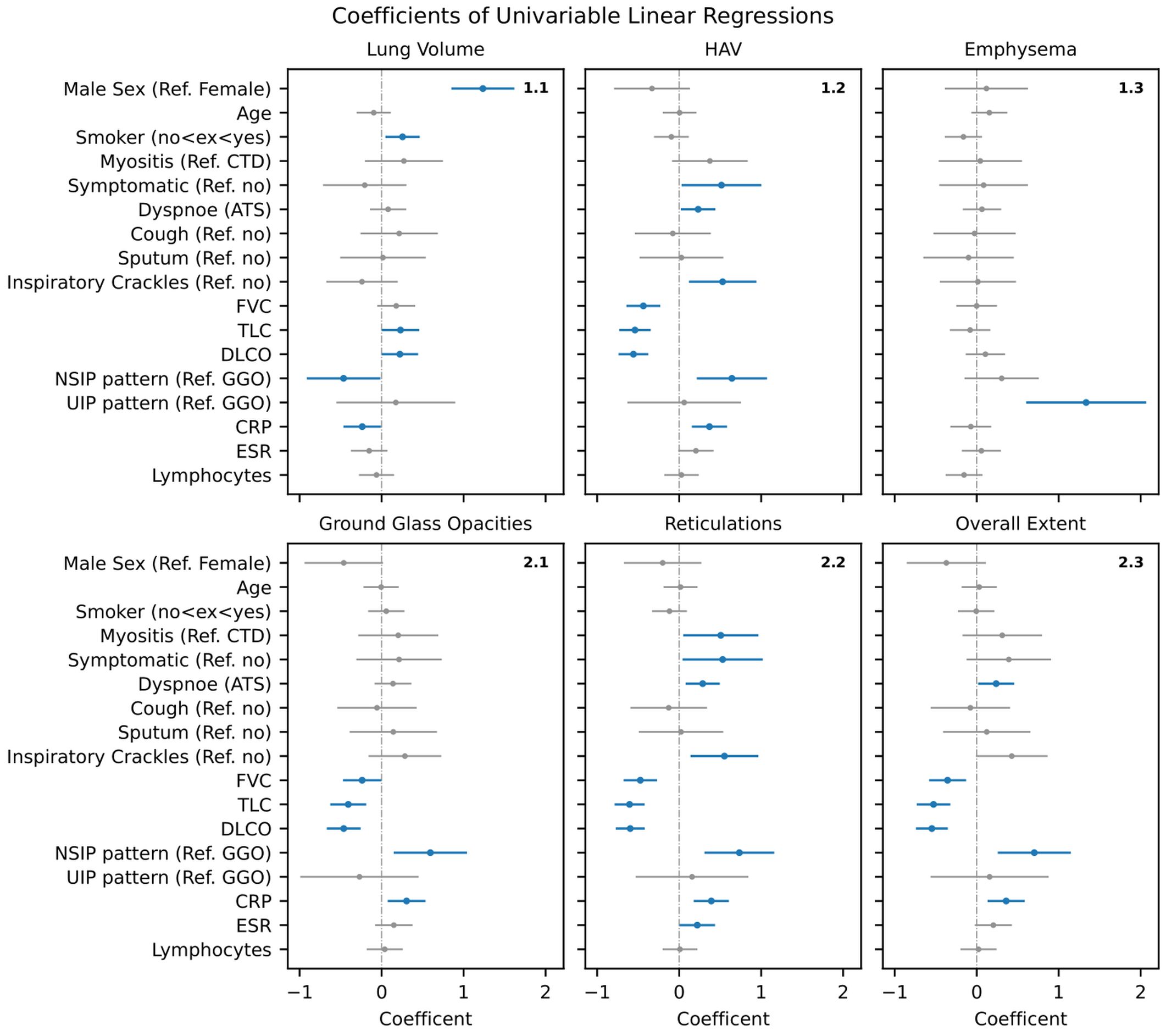

Supplement: online supplemental figure 1 [file rmdopen-11-4-s001.jpg]
