# Supplementary material for: AI-based HRCT quantification reveals DLCO and TLC as key determinants of ILD severity in connective tissue diseases
Source: RMD Open. 2025 Oct 28;11(4):e005963. doi: 10.1136/rmdopen-2025-005963 (PMC12570930; doi:10.1136/rmdopen-2025-005963)
Supplement: online supplemental file 1 [file rmdopen-11-4-s002.docx]

**Supplement:**

**Table 1:** Data of univariable linear regression of AIqpHRCT data for several clinical parameters (CI – confidence interval).

| **AIqpHRCT data** | **Variable** | **coefficient** | **Lower CI** | **Upper CI** | **p-value** |
| --- | --- | --- | --- | --- | --- |
| **Volume** | Gender | 1,23 | 0,85 | 1,62 | 0,00 |
|  | Age | -0,10 | -0,31 | 0,11 | 0,36 |
|  | Smoker status | 0,26 | 0,05 | 0,46 | 0,02 |
|  | Disease | 0,27 | -0,20 | 0,75 | 0,26 |
|  | Symptomatic (in general) | -0,21 | -0,72 | 0,30 | 0,42 |
|  | Dyspnoe | 0,08 | -0,14 | 0,30 | 0,48 |
|  | Cough | 0,21 | -0,26 | 0,69 | 0,37 |
|  | Sputum | 0,02 | -0,51 | 0,54 | 0,95 |
|  | Bibasilar inspiratory crackles | -0,24 | -0,68 | 0,20 | 0,28 |
|  | FVC (%) | 0,18 | -0,05 | 0,41 | 0,13 |
|  | TLC (%) | 0,23 | 0,00 | 0,46 | 0,05 |
|  | DLCO (%) | 0,22 | 0,00 | 0,44 | 0,05 |
|  | NSIP pattern (Ref: GGO) | -0,46 | -0,91 | -0,02 | 0,04 |
|  | UIP pattern (Ref: GGO) | 0,17 | -0,55 | 0,90 | 0,64 |
|  | CRP | -0,24 | -0,47 | -0,01 | 0,04 |
|  | ESR | -0,15 | -0,38 | 0,07 | 0,18 |
|  | Lymphocytes | -0,06 | -0,28 | 0,15 | 0,56 |
| **HAV** | Gender | -0,33 | -0,79 | 0,13 | 0,16 |
|  | Age | 0,00 | -0,20 | 0,21 | 0,96 |
|  | Smoker status | -0,10 | -0,31 | 0,12 | 0,37 |
|  | Disease | 0,37 | -0,09 | 0,84 | 0,11 |
|  | Symptomatic (in general) | 0,52 | 0,03 | 1,00 | 0,04 |
|  | Dyspnoe | 0,23 | 0,02 | 0,44 | 0,03 |
|  | Cough | -0,08 | -0,54 | 0,39 | 0,74 |
|  | Sputum | 0,03 | -0,48 | 0,54 | 0,91 |
|  | Bibasilar inspiratory crackles | 0,53 | 0,12 | 0,94 | 0,01 |
|  | FVC (%) | -0,44 | -0,64 | -0,23 | 0,00 |
|  | TLC (%) | -0,54 | -0,73 | -0,35 | 0,00 |
|  | DLCO (%) | -0,56 | -0,74 | -0,38 | 0,00 |
|  | NSIP pattern (Ref: GGO) | 0,64 | 0,21 | 1,07 | 0,00 |
|  | UIP pattern (Ref: GGO) | 0,06 | -0,63 | 0,75 | 0,86 |
|  | CRP | 0,37 | 0,15 | 0,58 | 0,00 |
|  | ESR | 0,20 | -0,01 | 0,42 | 0,06 |
|  | Lymphocytes | 0,03 | -0,18 | 0,24 | 0,79 |
| **Emphysema** | Gender | 0,12 | -0,39 | 0,63 | 0,64 |
|  | Age | 0,15 | -0,07 | 0,37 | 0,17 |
|  | Smoker status | -0,16 | -0,39 | 0,06 | 0,16 |
|  | Disease | 0,04 | -0,46 | 0,55 | 0,86 |
|  | Symptomatic (in general) | 0,08 | -0,46 | 0,62 | 0,76 |
|  | Dyspnoe | 0,06 | -0,17 | 0,30 | 0,59 |
|  | Cough | -0,03 | -0,53 | 0,47 | 0,92 |
|  | Sputum | -0,10 | -0,65 | 0,45 | 0,72 |
|  | Bibasilar inspiratory crackles | 0,01 | -0,45 | 0,48 | 0,95 |
|  | FVC (%) | 0,00 | -0,25 | 0,25 | 0,99 |
|  | TLC (%) | -0,08 | -0,33 | 0,17 | 0,52 |
|  | DLCO (%) | 0,11 | -0,13 | 0,35 | 0,38 |
|  | NSIP pattern (Ref: GGO) | 0,30 | -0,15 | 0,76 | 0,18 |
|  | UIP pattern (Ref: GGO) | 1,33 | 0,60 | 2,07 | 0,00 |
|  | CRP | -0,07 | -0,32 | 0,18 | 0,56 |
|  | ESR | 0,06 | -0,18 | 0,30 | 0,64 |
|  | Lymphocytes | -0,15 | -0,38 | 0,07 | 0,17 |
| **GGO** | Gender | -0,46 | -0,94 | 0,02 | 0,06 |
|  | Age | -0,01 | -0,22 | 0,21 | 0,95 |
|  | Smoker status | 0,06 | -0,17 | 0,28 | 0,61 |
|  | Disease | 0,20 | -0,29 | 0,69 | 0,41 |
|  | Symptomatic (in general) | 0,21 | -0,31 | 0,73 | 0,42 |
|  | Dyspnoe | 0,14 | -0,09 | 0,36 | 0,22 |
|  | Cough | -0,06 | -0,54 | 0,43 | 0,82 |
|  | Sputum | 0,14 | -0,39 | 0,67 | 0,60 |
|  | Bibasilar inspiratory crackles | 0,28 | -0,16 | 0,73 | 0,21 |
|  | FVC (%) | -0,24 | -0,47 | 0,00 | 0,05 |
|  | TLC (%) | -0,41 | -0,63 | -0,19 | 0,00 |
|  | DLCO (%) | -0,46 | -0,67 | -0,26 | 0,00 |
|  | NSIP pattern (Ref: GGO) | 0,59 | 0,15 | 1,04 | 0,01 |
|  | UIP pattern (Ref: GGO) | -0,27 | -0,99 | 0,45 | 0,46 |
|  | CRP | 0,30 | 0,07 | 0,53 | 0,01 |
|  | ESR | 0,15 | -0,08 | 0,38 | 0,20 |
|  | Lymphocytes | 0,04 | -0,18 | 0,26 | 0,73 |
| **Reticulations** | Gender | -0,20 | -0,67 | 0,27 | 0,40 |
|  | Age | 0,02 | -0,19 | 0,22 | 0,88 |
|  | Smoker status | -0,12 | -0,33 | 0,09 | 0,27 |
|  | Disease | 0,51 | 0,05 | 0,97 | 0,03 |
|  | Symptomatic (in general) | 0,53 | 0,04 | 1,02 | 0,03 |
|  | Dyspnoe | 0,29 | 0,08 | 0,50 | 0,01 |
|  | Cough | -0,13 | -0,59 | 0,34 | 0,59 |
|  | Sputum | 0,02 | -0,49 | 0,54 | 0,93 |
|  | Bibasilar inspiratory crackles | 0,55 | 0,14 | 0,97 | 0,01 |
|  | FVC (%) | -0,47 | -0,68 | -0,27 | 0,00 |
|  | TLC (%) | -0,61 | -0,79 | -0,42 | 0,00 |
|  | DLCO (%) | -0,60 | -0,77 | -0,42 | 0,00 |
|  | NSIP pattern (Ref: GGO) | 0,73 | 0,31 | 1,16 | 0,00 |
|  | UIP pattern (Ref: GGO) | 0,16 | -0,53 | 0,84 | 0,65 |
|  | CRP | 0,39 | 0,18 | 0,61 | 0,00 |
|  | ESR | 0,22 | 0,00 | 0,44 | 0,05 |
|  | Lymphocytes | 0,01 | -0,20 | 0,22 | 0,93 |
| **Overall extent of ILD** | Gender | -0,37 | -0,85 | 0,11 | 0,13 |
|  | Age | 0,03 | -0,18 | 0,24 | 0,78 |
|  | Smoker status | -0,01 | -0,23 | 0,22 | 0,96 |
|  | Disease | 0,31 | -0,17 | 0,80 | 0,20 |
|  | Symptomatic (in general) | 0,39 | -0,12 | 0,91 | 0,13 |
|  | Dyspnoe | 0,24 | 0,02 | 0,46 | 0,04 |
|  | Cough | -0,08 | -0,56 | 0,41 | 0,75 |
|  | Sputum | 0,12 | -0,41 | 0,65 | 0,65 |
|  | Bibasilar inspiratory crackles | 0,43 | -0,01 | 0,86 | 0,06 |
|  | FVC (%) | -0,36 | -0,58 | -0,13 | 0,00 |
|  | TLC (%) | -0,53 | -0,73 | -0,32 | 0,00 |
|  | DLCO (%) | -0,55 | -0,74 | -0,35 | 0,00 |
|  | NSIP pattern (Ref: GGO) | 0,70 | 0,26 | 1,15 | 0,00 |
|  | UIP pattern (Ref: GGO) | 0,16 | -0,56 | 0,88 | 0,67 |
|  | CRP | 0,36 | 0,13 | 0,58 | 0,00 |
|  | ESR | 0,20 | -0,02 | 0,43 | 0,08 |
|  | Lymphocytes | 0,02 | -0,20 | 0,24 | 0,85 |

**Figure 1:** Univariable linear regression of AIqpHRCT data for several clinical parameters (blue -marked parameters with significant effect in analysis).
